# The Importance of Sensory Processing in Mental Health: A Proposed Addition to the Research Domain Criteria (RDoC) and Suggestions for RDoC 2.0

**DOI:** 10.3389/fpsyg.2019.00103

**Published:** 2019-02-05

**Authors:** Laura A. Harrison, Anastasiya Kats, Marian E. Williams, Lisa Aziz-Zadeh

**Affiliations:** ^1^USC Chan Division of Occupational Science and Occupational Therapy, University of Southern California, Los Angeles, CA, United States; ^2^Brain and Creativity Institute, University of Southern California, Los Angeles, CA, United States; ^3^Children’s Hospital Los Angeles, University of Southern California, Los Angeles, CA, United States

**Keywords:** sensory processing, sensory perception, Research Domain Criteria (RDoC), sensorimotor, mental health, sensory processing disorder, autism spectrum disorder (ASD), schizophrenia

## Abstract

The time is ripe to integrate burgeoning evidence of the important role of sensory and motor functioning in mental health within the National Institute of Mental Health’s [NIMH] Research Domain Criteria [RDoC] framework (National Institute of Mental Health, n.d.a), a multi-dimensional method of characterizing mental functioning in health and disease across all neurobiological levels of analysis ranging from genetic to behavioral. As the importance of motor processing in psychopathology has been recognized (Bernard and Mittal, 2015; Garvey and Cuthbert, 2017; National Institute of Mental Health, 2019), here we focus on sensory processing. First, we review the current design of the RDoC matrix, noting sensory features missing despite their prevalence in multiple mental illnesses. We identify two missing classes of sensory symptoms that we widely define as (1) sensory processing, including sensory sensitivity and active sensing, and (2) domains of perceptual signaling, including interoception and proprioception, which are currently absent or underdeveloped in the perception construct of the cognitive systems domain. Then, we describe the neurobiological basis of these psychological constructs and examine why these sensory features are important for understanding psychopathology. Where appropriate, we examine links between sensory processing and the domains currently included in the RDoC matrix. Throughout, we emphasize how the addition of these sensory features to the RDoC matrix is important for understanding a range of mental health disorders. We conclude with the suggestion that a separate sensation and perception domain can enhance the current RDoC framework, while discussing what we see as important principles and promising directions for the future development and use of the RDoC.

## Introduction

### Development of the Research Domain Criteria

Clinicians and researchers have long grappled with the challenge of categorizing, describing, and understanding mental disorders, with imperfect results. The *Diagnostic and Statistical Manual of Mental Disorders* (American Psychiatric Association [APA], 2013) is currently the standard tool used in the United States for diagnosing individuals for purposes of eligibility for health insurance funding for treatment, and for communication amongst mental health professionals. The DSM-5 is also widely used by researchers studying psychopathology and the effects of treatments. Nonetheless, researchers and clinicians alike have acknowledged shortcomings of the approach to classifying mental illness in the DSM-5, as well as other categorical diagnostic systems such as the *International Classification of Diseases*. Problems with these systems stem from the complexity and dimensional nature of mental health symptoms, contrasted with the necessity of simplifying in order to categorize these disorders. These challenges, carefully explicated by Clark et al. (2017), include largely arbitrarily decided divisions between normal and abnormal and between one disorder and another. For example, although many psychological phenomena are thought to exist along a continuum, in a categorical system, thresholds must be set in order to differentiate normal versus disordered behavior. Similarly, high rates of comorbidity occur because of the heterogeneity in symptoms across disorders and clinicians’ need to include multiple disorders in a diagnostic formulation in order to capture the complexity and overlapping symptoms occurring in the same individual.

Although the categorical approach to diagnosis remains useful in public health and clinical contexts, an alternative strategy is needed to advance the scientific understanding of mental illness and ultimately improve clinical outcomes. Research approaches that incorporate the dimensionality and heterogeneity of mental phenomena as well as incorporating neuroscience are essential to advance knowledge about the underlying etiology of mental illness, and to understand the phenomena of mental health and mental illness across the full range of human behavior. To this end, the National Institute of Mental Health introduced the Research Domain Criteria (RDoC), a multi-level research framework, to study mental illness in ways that are grounded in neuroscience and eschew *a priori* diagnostic categories. It facilitates cross-talk between scientists working at different levels of analysis in order to examine functioning in health and disease across a spectrum of individuals. The current RDoC design includes a matrix with six domains: (1) positive and (2) negative valence, (3) cognitive systems, (4) social processes, (5) arousal and regulatory systems, and, just added to the original five domains, (6) sensorimotor systems (National Institute of Mental Health, 2019). Positive and negative valence refers to responses to motivational and aversive situations; cognitive systems are associated with cognitive processes such as attention and memory; social processes concern interpersonal perceptions and interactions; arousal and regulatory systems initiate the activation of neural systems; and sensorimotor systems control the execution of motor actions (National Institute of Mental Health, n.d.a). The RDoC recognizes development and environmental influences as essential components across domains (National Institute of Mental Health, n.d.b), and specific suggestions have been made for incorporating developmental approaches into the RDoC framework (Casey et al., 2014; Mittal and Wakschlag, 2017).

### Sensory Holes in the RDoC

The RDoC was intended to be a dynamic, evolving tool, with a proposed process for recommending changes to the model. For example, a report recommending the addition of a motor domain was submitted to a workgroup of the National Advisory Mental Health Council (NAMHC), a group formed to oversee changes to the RDoC Matrix in conjunction with members of the NIH RDoC workgroup (Garvey and Cuthbert, 2017). This recommendation eventually led to the addition of a sensorimotor domain to the RDoC Matrix, announced during the typesetting of this article (National Institute of Mental Health, 2019). The development of the NAMHC workgroup as well as this recent change signal a sincere effort to update and revitalize the RDoC. Given that change is welcome, we propose that inclusion of a sensory processing domain will significantly benefit the utility of the RDoC for describing individual differences in behavior and functioning.

Looking at the current RDoC, sensory processing is primarily represented by the inclusion of (1) a perception construct within the cognitive systems domain; (2) some domain-specific categories of social perception within the social processes domain; and (3) from the new sensorimotor domain, the sensorimotor dynamics subconstruct of the motor actions construct whereby sensory input modulates actions plans, as well as the agency and ownership construct whereby one feels a sense of control for one’s body and volitional actions. Within the cognitive systems perception construct, visual and auditory perception are detailed across all units of analysis, ranging from implicated molecules to relevant paradigms. A third “olfactory/ somatosensory/multimodal/perception” subconstruct serves as a bookmarker for the remainder of our sensory experience, with only two paradigms, (1) “manipulation of ISI (interstimulus interval) and/or intensity” and (2) “smell identification,” being listed, and no other details included. Reviewing the current RDoC as a whole, it is notably missing core sensory symptoms commonly associated with a range of mental illness. These symptoms can be broadly described by two categories: (1) sensory processing, especially disruptions in sensory sensitivity and active sensing, and (2) perceptual sensory signals currently either missing from or underrepresented in the RDoC, notably including both interoceptive and proprioceptive cues. We propose that these constructs be added to the RDoC, potentially within a new sensory processing domain.

The RDoC website (National Institute of Mental Health, n.d.b) details three criteria for proposed changes. These are the same criteria that were used for including a new construct during the initial development of the RDoC. In this review, we aim to address these criteria with respect to our two proposed constructs: sensory processing and an expanded set of sensory perceptual signals. The first criteria is that a psychological construct or functional behavior must be implicated, while the second criteria is that there must be evidence for an implicated neural system or circuit that underlies that behavioral function. In response to these first two criteria, we begin our review of each of the two proposed sensory constructs with an overview of the relevant psychological constructs as well as implicated neural structures. Finally, the third criteria is that the construct must relate to a clinically-relevant symptom. To that end, we provide examples of sensory dysfunction across a range of mental disorders and clinical symptoms. In addition to these criteria, the construct should be able to be investigated across the units of analysis detailed in the RDoC, ranging from cellular and genetic mechanisms to behavior; thus, we provide evidence for each construct spanning several units of analysis.

## Sensory Processing

### Sensory Processing: Definition, Measurement, and Neurobiological Basis

Sensory perception mediates our interaction with the world, but our sensory experience is more nuanced than simple perception. It is useful to consider sensory perception and sensory processing separately. Sensory processing implicates domain-general processes, which may generalize across specific perceptual modalities. Two types of sensory processing disruptions will be discussed: abnormalities in sensory sensitivity, and disruptions in active sensing or sensori-motor loops. Disruptions in sensory sensitivity have a long history of description in psychology and occupational science (Baranek et al., 2013), among other fields, at the level of symptoms, with emerging work describing the biological underpinnings of those symptoms. Although disruptions in active sensing or sensori-motor loops may eventually be included within a motor domain in the RDoC, they are currently missing from the RDoC and are an important and fundamental aspect of sensory processing across species. Before discussing how these two disruptions in sensory processing relate to mental illness, we first further define these constructs, their measurement, and neurobiological basis below.

#### Sensory Sensitivity

Sensory sensitivity can be described as “the degree to which one is susceptible to perceiving small changes in stimulus intensity” (Schauder and Bennetto, 2016). Sensory sensitivity can be, but is not limited to, the result of abnormalities in sensory processing (the ability to receive and respond to stimuli), gating (inhibitory functioning at the neural level, which filters out redundant information), habituation (decreased neural response to repetitive sensory stimuli), and/or cross-modality (perception between two or more sensory modalities).

Much research in relation to sensory processing has been completed in fields focusing on clinical symptoms, and much bridging research is needed to relate those findings to neuroscience. Schauder and Bennetto (2016) provide an example of this bridging work in their review of sensory dysfunction in autism spectrum disorder (ASD). The authors relate terms from the sensory literature to terms from the neural literature, and then conceptually map these concepts to different levels of analysis, ranging from neural brain structure at one extreme to clinical symptom at the other. Examples are provided of experimental methods and findings at each level of analysis relevant to the common clinical case of auditory hyper-responsivity. Regarding terms, some are shared by both the neural and symptoms literature, albeit with a different focus on whether neural or behavioral responding is referenced: behavior, low threshold, high threshold, hyper-responsiveness, and hypo-responsiveness are terms used by both literatures. Sensory gating is specific to the neural literature, while symptom, defensiveness, avoidance, poor registration, sensory orienting, sensory filtering, and sensory seeking are specific to the symptom literature. Many of these sensory terms describe behaviors that may represent several cognitive and perceptual processes. For example, poor registration is the decreased ability to “register” sensory input and is usually measured by a lack of response to the stimulus. Several processes may underlie this behavioral symptom. Bringing tools from cognitive psychology, such as signal detection theory, to sensory processing research (Gallun and Kampel, 2017), one could isolate whether difficulties in registration were a result of an inability to detect a stimulus, or simply a contextual tendency to not respond to a stimulus, perhaps due to attentional or motivational constraints. Indeed, there are several top–down influences on sensory processing; we refer the reader to Gilbert and Sigman (2007) for a comprehensive review.

The importance of sensory sensitivity is also underwritten by recent trends in the field of computational psychiatry in which computational and theoretical methods are applied to psychopathology (Friston et al., 2014; Wang and Krystal, 2014). Sensory processing is indeed an apt target for this field, as basic sensory processing was an early target of the antecedent field of computational neuroscience (Sejnowski et al., 1988). An emerging theme in computational psychiatry in relation to sensory sensitivity is a failure of sensory attenuation — usually cast in terms of a failure to modulate the precision of sensory prediction errors within a predictive coding framework. Psychophysically, this translates directly into a failure to modulate sensory sensitivity, and has been proposed as a key etiological factor in many conditions, ranging from autism to schizophrenia and from depression to chronic pain disorders (Pellicano and Burr, 2012; Adams et al., 2013; Van de Cruys et al., 2014; Powers et al., 2017; Clark et al., 2018; Hoskin et al., 2019), some of which we will further explore below.

While thus far, we have focused on mechanisms that relate to higher-order units of analysis in the RDoC, it is important to recognize the perhaps obvious fact that neuromodulators can modulate sensory sensitivity. For example, in the case of visual perception, contrast sensitivity of V1 neurons in the striate cortex was found to be reduced following muscarinic and nicotinic receptor blockade in awake, behaving macaques, implicating acetylcholine in visual signal enhancement (Herrero et al., 2017). Spanning several sensory systems, the monoamine neuromodulators serotonin, dopamine, and noradrenaline modulate several aspects of sensory processing across sensory modalities (Jacob and Nienborg, 2018). Pertinent to the computational view, cholinergic neurotransmission is thought to play a key role in sensory attenuation and the setting of precision within a predictive coding framework (Vinogradova et al., 1996; Yu and Dayan, 2005; Parr and Friston, 2017). Recent theoretical and simulation research (Parr et al., 2018) indicates that such cholinergic signaling of sensory precision may occur within a biologically plausible Bayes-optimal framework whereby updating of prior beliefs based on newly acquired sensory evidence occurs through gradient descent on variational free energy. Updating of sensory beliefs is relevant to the concept of active sensing, discussed further below. Given the role of neuromodulators in sensory coding, sensory processing can be studied from the molecular to the systems level, making it appropriate for the RDoC’s framework of understanding a clinical symptom across different units of analysis.

#### Active Sensing and Sensory-Motor Loops

Although sensory perception diagrams often detail the passive perception of an isolated sensory stimulus, in practice, sensory perception is a dynamic process, involving both the processing and filtering of continuously received sensory signals into a coherent percept, as well as requiring coordination of motor actions that may result in actively acquiring additional sensory input (cf., Nelson and MacIver, 2006 for a review of active sensing systems). For example, to increase sampling of both time and place, mice may employ ongoing control of moving their whiskers over an object to increase sensory processing (Deutsch et al., 2012). Non-linear processing of sensory and motor signals in apical tuft dendrites in layer 5 pyramidal neurons of the barrel cortex may underlie active sensing through such whisking (Xu et al., 2012). Even in plants, which have limited motor capacity, it is well accepted that leaves and branches move toward a light source. The specific neurobiological basis of active sensing has been explored with respect to visual drifts and microsaccades (Ahissar et al., 2016) and even miniature eye movements during fixation (Ahissar and Arieli, 2012); the categorizing of visual patterns (Yang et al., 2016); the biomechanics of licking and taste qualities in mice (Graham et al., 2014); the combined role of motor and sensory processing in human tactile perception (Saig et al., 2012) and rodent perception of object location (Saraf-Sinik et al., 2015). Motor rhythms have been proposed as a general mechanism enabling various forms of active sensing (Schroeder et al., 2010). On the computational psychiatry view, formulations of psychopathology in terms of active inference rest explicitly on active sensing. For example, the slow pursuit eye movements that characterize schizophrenia (Adams et al., 2013) or the role of dopamine in attenuation of proprioceptive sensations in Parkinson’s disease (Oestreich et al., 2015; Wiese, 2017).

Clearly, motor and sensory processing are often entangled together. It makes sense, then, that motor processing is often associated with abnormal sensory processing in many clinical disorders. However, although some sensory processing interacts with motor processing, much of sensory processing is independent of these sensory-motor loops, and sensory processing should still be considered as a potential addition to the RDoC framework.

### Sensory Sensitivity in Health and Disease

Sensory sensitivity plays an important role in sensory processing sensitivity (SPS), a personality trait, as well as in the clinical symptomatology of sensory processing in ASD, sensory processing disorder (SPD), depression, and anxiety disorders, as we further discuss below.

#### Sensory Processing Sensitivity

Sensory processing sensitivity is defined as a genetically determined personality trait described by “social, emotional, and physical sensitivity” (Jagiellowicz et al., 2010; Aron et al., 2012). As studied by Jagiellowicz et al. (2010), variation in this trait is associated with neurobiological differences. When individuals were shown images with subtle alterations, such as adding half a hay bale to the haystack in the previous image, those high in SPS had a greater degree of activation of brain regions involved in high-order visual processing (Jagiellowicz et al., 2016). Another study by Acevedo et al. (2014) found a positive correlation between an individual’s Highly Sensitive Person score, a standardized survey to test for SPS, and activation of brain regions involved in attention, empathy, and action planning.

It should be noted that SPS has overlapping features with sensory processing abnormalities present in disorders such as ASD, schizophrenia, and post-traumatic stress disorder (PTSD). Acevedo et al. (2018) explored how SPS was different than clinical disorders marked by sensory processing abnormalities. The study reviewed fMRI data from studies including participants with SPS, ASD, schizophrenia, and PTSD. They found that all four groups had activation of the precentral gyrus, but SPS was the only group to show activation of the ventral tegmental area and substantia nigra only in response to positive stimuli; hypothalamus, periaqueductal gray, insula, inferior frontal gyrus, temporoparietal junction, and prefrontal cortex in response to social and emotional stimuli (Acevedo et al., 2018). These findings suggest that SPS can be studied independently of the other conditions as well as across the spectrum of health, aligning with the goals of the RDoC framework.

#### Sensory Processing Disorder (SPD)

Sensory processing disorder is defined by abnormalities in the modulation and processing of stimuli that interferes with daily functioning (Miller et al., 2009). However, it should be noted that there is controversy over whether SPD is a distinct disorder or if abnormal sensory processing is a characteristic of other disorders (Zimmer et al., 2012). The American Academy of Pediatrics recommends that physicians should not diagnose individuals experiencing problems with sensory processing as having SPD; rather they should consider developmental disorders such as ASD, developmental coordination disorder (DCD), and anxiety disorder (Zimmer et al., 2012). However, SPD is included in Diagnostic Classification of Mental Health and Developmental Disorders of Infancy and Early Childhood, the Diagnostic Manual for Infancy and Early Childhood of the Interdisciplinary Council on Developmental and Learning Disorders, and the Psychodynamic Diagnostic Manual (Miller et al., 2007). Whether it is considered a disorder or not, there exists evidence that abnormal sensory processing results in neurobiological differences, which are relevant to the RDoC approach. Owen et al. (2013) found a correlation between reduced white matter microstructure (mostly affecting the posterior cerebral tract) and abnormal sensory integration between individuals with SPD and typically developing children. On the other hand, those with ADHD have reduced white matter primarily associated with their fronto-striatal pathways, while those with ASD have altered white matter tracts affecting mostly the corpus callosum, cingulum, and areas of the temporal lobe (Tamm et al., 2012; Travers et al., 2012). Such white matter differences may prove to be a useful biomarker. Furthermore, since sensory abnormalities occur commonly in mental health disorders, we can begin to use sensory processing as a means for differentiating between and within disorders through this biomarker in order to create more homogeneous subgroups of disorders, which is especially useful in research. A study done by Tavassoli et al. (2018) did just that. They explored the differences in symptomatology between those with ASD and sensory processing disorder. They found that both groups exhibited sensory symptoms, however there was a negative correlation between the number of sensory symptoms and the level of empathy. This suggests as we consider symptoms of disorders and their associated brain areas, we may have to look at multiple domains and their intersection to characterize a disorder.

The American Academy of Pediatrics goes on further to recommend that physicians should inform parents about the minimal evidence to support sensory based therapies, a type of therapy typically used by occupational therapists in order to regulate a child’s response to external stimuli (Zimmer et al., 2012). However, if we expand our knowledge of sensory processing by including it in the RDoC, researchers can potentially develop effective therapies targeting abnormal responses to stimuli.

#### Sensory Sensitivity in Autism Spectrum Disorder (ASD)

Autism spectrum disorder is a neurodevelopmental disorder characterized by social communication deficits, restricted interests, and repetitive behaviors. One of the symptom criteria for ASD in the DSM-5 is “hyper- or hypo-reactivity to sensory input or unusual interest in sensory aspects of the environment” (American Psychiatric Association [APA], 2013, p. 50). This symptom was added to the DSM based on findings from multiple studies that both children and adults with ASD commonly exhibit sensory sensitivities that result in under-responsiveness and/or over-responsiveness (Ben-Sasson et al., 2007; Crane et al., 2009; Cermak et al., 2010; Marco et al., 2011; Baranek et al., 2013; Robertson and Simmons, 2013). In a study of youth with ASD, Green et al. (2015) found a positive correlation between over-responsiveness and greater activation of primary sensory cortices and the amygdala, indicating a neurobiological basis for sensory sensitivity.

It has been hypothesized that sensory sensitivities may contribute to or even be a primary symptom of ASD (Donnellan et al., 2013). For instance, social communication relies on one’s ability to perceive, integrate and appropriately respond to information received through the senses, such as subtleties of posture, eye contact, and intonation (Hannant et al., 2016). Thus, impairments in sensory processing, such as seen in individuals with ASD, can lead to difficulties with communication. This barrier may result in more social communication deficits as seen in the positive correlation between the number of sensory sensitivities and severity of ASD in children (Kern et al., 2007; Hannant et al., 2016). Another study by Hannant et al. (2018) found that visual and auditory sensitivities in ASD and proprioception sensitivity in DCD contributed to movement abnormalities seen in these disorders. Thus, understanding sensory sensitivities and which modality they stem from (auditory, visual, proprioception) may be important for better understanding and treating clinical disorders as well as distinguishing between them. For instance, when treating motor abnormalities in an individual with ASD, we may be more effective in implementing treatment that targets visual and auditory sensitivity specifically.

Additionally, sensorimotor abnormalities may be one of the first observable signs of ASD (Levit-Binnun et al., 2013). Therefore, in addition to current diagnostic tools that rely heavily on social skills, focusing on sensory processing can help clinicians more reliably diagnose individuals at a younger age. For example, ASD is typically diagnosed around 3 to 4 years of age despite the presence of atypical social behavior appearing between 6 and 12 to 18 months (Zwaigenbaum et al., 2015). This is because social behaviors, such as limited eye contact and abnormal social play, are difficult to observe and/or characterize at such a young age (Teitelbaum et al., 1998; Baranek, 1999). On the other hand, sensory abnormalities may be present from as early as 12 months, and resulting behaviors such as abnormal visual fixation, are easily recognizable and can be recorded via home videos and pictures (Bryson et al., 2007). Thus, parents can bring this information to clinicians at an earlier age and begin treatment sooner, which is crucial as earlier intervention is associated with more positive treatment (Wallace and Rogers, 2010).

#### Sensory Sensitivity in Depression and Anxiety

Two of the most common mental illnesses, which often occur comorbidly, are depression and anxiety disorders (Hunt et al., 2002). A positive correlation between SPS scores and the presence of depression and/or anxiety has been found (Liss et al., 2005). However, others have found sensory symptoms only in individuals with anxiety disorder and not depression (Neal et al., 2002). Further research will need to be done to understand the presence and role of altered sensory processing role plays in each disorder, but it is a link worth exploring.

#### Sensory Sensitivity in Obsessive-Compulsive Disorder (OCD)

Obsessive-compulsive disorder (OCD) is characterized by *obsessions* — recurrent and persistent thoughts — and *compulsions* — repetitive behaviors or mental acts performed to avoid anxiety — whose presence causes distress, is time-consuming, and/or impairs functioning. Sensory processing differences have been proposed in both adults and children with OCD. Consistent with hypothesized differences in OCD, increased sensory sensitivity and sensation avoiding behavior, as indexed by the Adolescent/Adult Sensory Profile (AASP), were observed in 51 adults with OCD relative to a normative adult population (Rieke and Anderson, 2009). More frequent low registration and less frequent sensation seeking behavior were also observed in this adult OCD sample, albeit with a smaller effect size than the hypothesized increases in sensory sensitivity and sensation avoiding behavior. The non-hypothesized changes were potentially explained by comorbidities (e.g., depression, other anxiety disorders, and attention deficit disorders) and medication use (e.g., SSRIs) in the study sample as well as by a potential of the AASP to capture motivational as well as sensory aspects of self-reported behavior (Rieke and Anderson, 2009). Sensory intolerances have also been reported as a primary symptom of pediatric OCD (Hazen et al., 2008): in case studies of six children, compulsions were performed not in response to obsessions, but rather to relieve intolerance and intrusive re-experiencing of sensory stimuli from several sensory modalities (auditory, tactile, olfactory, and visual). In these patients, sensory phenomena (SP) were the primary presenting symptom and predominant source of distress (rather than compulsions or obsessions). Even when controlling for trait anxiety, (1) sensory over-responsivity is linked to childhood ritualism, and (2) increased oral and tactile hypersensitivity recalled from childhood is related to increased OCD symptoms in adults, suggesting that hypersensitivity and ritualism in childhood relate to at least a subtype of adult OCD (Dar et al., 2012).

A common theme in these pediatric reports is that ritualistic behaviors are developed in response to abnormal sensory experience in order to create control over one’s sensory environment. Reports of similar sensory abnormalities, or SP are also commonly reported in adults, especially those with pediatric OCD onset, and include such subjective experiences as various bodily sensations and urges to pursue “just right” perceptions, as well as sensory tics (do Rosário et al., 2017). While not unique to OCD, and not always considered central to OCD, SP *are* commonly recognized when OCD patients are directly questioned about symptoms; therefore, it has been suggested that SP should be considered a core component of OCD (do Rosário et al., 2017).

### Active Sensing and ASD

Sensorimotor integration, which requires coordination of action and perception, is often implicated in ASD (Cattaneo et al., 2007; Paton et al., 2012) and is frequently noted as a core feature of ASD (Robledo et al., 2012). Scientifically, sensory and movement differences have been proposed as a fundamental rather than secondary feature of autism (Donnellan et al., 2013; Savarese, 2013; Whyatt and Craig, 2013), and are key targets of occupational therapy for the disorder (Case-Smith and Arbesman, 2008). Sensorimotor integration is closely related to the concept of perception-action or active sensing. It has been proposed that disruptions of these abilities in ASD children may be related to disruptions in the mirror neuron system (MNS) as well as in cortico-cerebellar loops (Von Hofsten and Rosander, 2012). Specifically, it is proposed that the MNS (also referred to as the Action Observation Network or AON in human neuroimaging literature), which is involved in both the perception and execution of motor actions, generates predictive models of the sensory consequences of action through parietal-premotor connections, and through a simulation account are posited to play an important role in interpreting the intentions of others’ actions (Dapretto et al., 2006; Von Hofsten and Rosander, 2012). As such, disruption in the MNS/AON may manifest in both sensorimotor and social difficulties (empathizing and intention understanding/mentalizing) as is observed in autism. Meanwhile, disruptions in the cortico-cerebellar loop from the posterior parietal cortex (PCC) to the cerebellum, back to motor/premotor areas of the cortex may relate to motor-action prediction problems in ASD (Von Hofsten and Rosander, 2012). Such prediction problems will directly relate to disturbances in sensorimotor integration and active sensing.

In typically developing children, haptic perception — the active sensing of object surfaces through palpation — is a skill developed throughout development, progressing from relatively unguided exploratory movement early in life to adult-level haptic acuity observed in early adolescents (Gori et al., 2012). Haptic perception involves the production of efference copies for guiding action; these efference copies must be integrated with sensory feedback, including proprioceptive and kinematic feedback on the location and movement of limbs as well as mechanosensory feedback regarding the palpated object (see below for a discussion of specific sensory perception modalities). In development, these efference copies are thought to be noisy, resulting in a poor model of predicted sensory feedback and poor haptic acuity (Gori et al., 2012). If proprioceptive feedback is also poor, as may be the case in autism (Haswell et al., 2009), the process will be further disrupted.

Beyond haptic perception, the active acquisition of visual stimuli may be affected in autism. A large literature explores abnormal eye gaze in autism, including disturbances in where people fixate or look when viewing both socially and non-socially relevant stimuli. Attentional and motivational mechanisms may underlie such differences. However, there is also evidence that there may be disruption in the acquisition of visual information in autism: abnormal visually-guided eye movements or saccades have been observed in autism and it has been proposed that these differences may be related to cerebellar dysfunction (Johnson et al., 2012).

## Sensory Perception in Mental Health

In addition to the general disruptions in sensory processing discussed above, the inclusion of a wider range of specific sensory modalities would also benefit the RDoC framework. While the RDoC does include some elements of sensory perception within the perception construct of the cognitive domain, it focuses disproportionally on audition and vision, while leaving other forms of sensory processing underdeveloped. Below we describe two major divisions in sensory processing — interoception and exteroception — and detail different exteroceptive sensations. Within this framework, we provide examples, certainly not exhaustive, of how sensations beyond audition and vision are related to mental and physical health.

### Divisions in Sensory Perception: Interoception and Exteroception

One major division exists within sensory processing: interoceptive cues come from within the body, while exteroceptive cues originate externally (Craig, 2002, 2003; Quadt et al., 2018). Certain sensations, such as proprioception, the sense of the position of one’s body in the environment, are intermediate, relying on both external and internal cues, but ultimately are concerned with relaying the state of the external world, albeit the body’s position within it. Interoceptive cues are crucial to maintaining basic homeostasis, guiding such basic drives as hunger and thirst, and enabling survival. Exteroceptive sensations can also have important adaptive value — as illustrative examples, consider the importance of nociception, the perception of pain, to recoiling from a damaging stimulus, or olfaction to detecting smoke, or gustation to rejecting a bitter poison. Disruptions in sensory processing across the external/internal division can lead to disruptions in other cognitive processes, ranging from more internally focused (valuation, learning, memory) to externally focused (attention), such that interoceptive or exteroceptive processing can affect all levels of cognition, contributing to a myriad of manifestations of disruption in mental disorders. While here we investigate disruptions in interoception and exteroception separately, following the taxonomy detailed in Table 1, an interesting future avenue of research is investigating the impact of disruptions in interoception on exteroception and vice versa.

**Table 1:** Taxonomy of sensations, related neural circuits, and associated mental disorders.

Sense	Categories	Sub-category	Neural circuits	Associated disorders
**Interoception** Sensations from internal signals			Anterior cingulate cortex, insula (cf. Craig, 2002, 2003), organum vasculosum, subfornical organ, thalamus, hypothalamus, periaqueductal gray, parabrachial nucleus, nucleus of solitary tract, ventrolateral medulla, area postrema, vagus nerve (Quadt et al., 2018); amygdala (Knuepfer et al., 1995)	Panic disorder (Ehlers and Breuer, 1992; Ehlers, 1993), depression (Avery et al., 2014), post- traumatic stress disorder (Glenn et al., 2016), generalized anxiety disorder (Pollatos et al., 2007), autism spectrum disorder (Garfinkel et al., 2016)
**Exteroception** Sensations from external stimuli	**Somatic Sensation** Sensations arising from the skin	**Mechanosensation** Sense the size, shape, and texture of objects as well as sense their movement across the skin	Dorsal root ganglion, lateral parabrachial nucleus, thalamus, cerebral cortex	Autism spectrum disorder (Tavassoli et al., 2016)
		**Nociception** Sense pain	Dorsal root ganglion, spinothalmic tract, thalamus, sensory cortex, limbic areas	Fibromyalgia (Serra et al., 2014), Post-traumatic stress disorder (Lerman et al., 2016)
		**Thermosensation** Sense temperature	Dorsal root ganglion, spinothalmic tract, lateral parabrachial nuclei, preoptic area, thalamus	Depression (Bär et al., 2005)
		**Proprioception^∗^** Sense of the static position and movement of the limbs and body	Dorsal root ganglion, primary sensorimotor cortex, thalamus, basal ganglia	Schizophrenia (Arnfred et al., 2015)
	**Chemical** Sensations arising from chemical substances	**Olfaction** Smell	Dorsal root ganglion, primary olfactory cortex, thalamus, orbito- frontal cortex, amygdala, hypothalamus	Alzheimer’s disease (Kesslak et al., 1988)
		**Gustation** Taste	Dorsal root ganglion, facial nerve, glossopharyngeal nerve, thalamus, gustatory cortex	Eating disorder (Dazzi et al., 2013)
	**Vision^∗∗^** Sight		Koniocellular, magnocellular, and parvocellular cells, cortio-cortical connections into supra- and infra- granular layers, superior colliculus, suprachiasmatic nucleus, retinal lateral interactions, top–down cortical interaction	Schizophrenia (Ford et al., 2014)
	**Audition^∗∗^** Hearing		Corticofugal, dorsal/ventral auditory streams	Schizophrenia (Ford et al., 2014)


*^∗^Proprioception requires processing of external and internal cues; it is included under the umbrella of exteroception because like the other somatic sensations, it involves dorsal root ganglion neurons*.

*^∗∗^Extensively covered in the RDoC; the neural circuits listed are taken directly from the RDoC.*

### Interoception

Where exteroception deals with the processing of external sensory information, interoception is defined as the processing of signals by internal bodily sensations. Specific definitions have varied over the centuries, with some being more inclusive, and some more restrictive (Ceunen et al., 2016). Recently, Critchley and Garfinkel (2018) defined interoception solely as the “afferent signaling of the internal states” whilst Khalsa et al. (2017) defines it as the processing of both afferent and efferent signals. Some definitions include only conscious processing of bodily states; others include unconscious body-brain signaling processes. Inconsistencies in the definition of interoception have made it difficult for scientists to collaborate and advance our knowledge of interoceptive processing. Such inconsistencies must be resolved and working definitions agreed upon (cf. Ceunen et al., 2016), yet are beyond the scope of this paper.

In the current paper, we will consider the impact of interoception on mental health following Quadt et al.’s (2018) more inclusive definition of interoception, which includes four components. The first component detailed by Quadt et al. (2018) are (1) body-to-brain signaling, which includes both neural and humoral signaling, such as immune and endocrine signaling. One relevant emerging body of research is that on brain-gut signaling, with several mechanisms of communication proposed between the enteric and central nervous systems (Mayer, 2011). The next two components of interoception are detailed as (2) the basic neural encoding of this body-to-brain information to represent and integrate several bodily signals at the neural level, and then (3) the influence of these neural representations of bodily state on other neural processes, ranging from perception, to cognition, to behavior (Quadt et al., 2018). Important for potential inclusion of interoception as a construct within the RDoC, these processes can be assessed across several units of analysis and involve interaction with constructs represented in the other domains — for example, one could investigate the relationship between (unconscious) neural sensitivity to some internal bodily signal and other abilities like empathic ability or social attention. Finally, the fourth component of interoception involves interoceptive feelings — the “psychological expression” of our neural representations of internal bodily states. These are our consciously accessible physical sensations or “feelings” (Quadt et al., 2018). Notably, only this fourth component requires consciousness, which should be taken into account as behavioral paradigms continue to be developed.

Interoception underlies the mind-body connection and impacts emotional, motor, and affective processing, which makes studying it essential for identifying abnormalities in brain functioning (Khalsa et al., 2017). Yet, researchers and clinicians have struggled to study interoceptive processes effectively because of the inconsistency in definition and taxonomy mentioned above and the multifaceted nature of interoception — it works across constructs relevant to several RDoC domains and different bodily systems, making it difficult to study in isolation (Khalsa et al., 2017).

However, new techniques and theories provide tools and a framework for feasibly studying interoception, making it an appropriate construct to be detailed in the RDoC. Khalsa et al. (2017) outlined several non-invasive and invasive techniques, including transcranial magnetic stimulation, transcutaneous vagus nerve stimulation, and intracranial electrode recordings, that have the potential to further understanding of interoception across several units of analysis.

Interoception is seen increasingly as a cornerstone of emotional inference — which may be related to depressive disorders — and, indeed, even a sense of the embodied self (Seth et al., 2012; Seth, 2013; Palmer et al., 2015; Stephan et al., 2016). The latter is particularly interesting in relation to notions of selfhood and dissociative conditions such as derealization and depersonalization. Other clinical disorders that may involve interoception deficits at various neurobiological levels include: ASD, PTSD, depression, generalized anxiety disorder (GAD), and panic disorder (PD) among others (cf. Table 1); below, we discuss how interoception relates to emotional experience and a subset of these disorders.

#### Interoception and Emotional Experience

Interoception affects multiple domains of functioning, including affective processing. Wiens et al. (2000) examined this relationship in a healthy population, testing whether heartbeat detection impacted how one experiences emotions. They found a positive correlation between interoceptive accuracy and the intensity of emotional experiences. This correlation will be interesting to explore further when considering disorders marked by heightened emotion, such as borderline personality disorder. Researchers can consider questions such as to what extent does interoception accuracy predict severity of symptoms in these disorders?

It should be noted that heartbeat detection is only one form of interoceptive testing and cannot encompass all aspects of interoception. Further research will need to be done to understand how the various components of interoception affect emotion intensity.

#### Interoception and Depression

Symptoms of depression are similar to those of behaviors indicating signs of physical illness (sickness behaviors) — lethargy, social withdrawal, and reduced movement (Dantzer et al., 2008). Some of these sickness behaviors may develop as a result of changes in brain regions associated with interoception. For instance, fatigue may be correlated with changes in the mid/posterior insula and left anterior cingulate (Harrison et al., 2009), regions implicated in interoceptive processing.

Some researchers have begun to explore interoceptive processing in individuals with depression. For example, compared to healthy controls, individuals with depression have decreased activation of dorsal mid-insula cortex (dmIC), a region of the brain involved in interoception and emotional control (Avery et al., 2014). Furthermore, there is increased resting-state functional connectivity between amygdala and dmIC. This aberrant connectivity has been positively correlated with depression severity (Avery et al., 2014). However, future studies will need to be done to understand whether abnormalities in interoception are the result or cause of emotional disturbances.

#### Interoception and ASD

As mentioned previously, interoception impacts emotional processing. Thus, disorders marked by disrupted emotional processing, such as ASD where individuals have difficulty recognizing their own and others’ emotions, may be marked by abnormalities in interoceptive processing (Bal et al., 2010). Garfinkel et al. (2016) found evidence in support of this theory: individuals with ASD had reduced interoceptive accuracy alongside heightened interoceptive sensitivity, using a self-report measure exploring how well an individual understands their bodily signals. These abnormalities were positively correlated with anxiety symptoms and difficulty identifying emotions, suggesting that abnormalities in interoception may be partly responsible for the primary symptoms associated with ASD (Garfinkel et al., 2016).

#### Interoception and Panic Disorder

Panic disorder is a type of anxiety disorder that is characterized by recurring unexpected panic attacks with symptoms such as heart palpitations, dyspnea, paresthesias, dizziness, and derealization. Hypervigilance to body symptoms is characteristic of PD. In some theories, interoception is thought to be central in the disorder, with individuals with PD experiencing increased interoceptive sensitivity (Ehlers and Breuer, 1992; Ehlers, 1993; Van der Does et al., 1997). Alternatively, inaccurate interpretations about feelings, such as catastrophic beliefs may be key. To better understand the relationship between two components of interoception — interoceptive sensibility (IS) and metacognitive interoception (MI) — and PD, Yoris et al. (2015) asked 21 individuals with PD and 12 controls to perform two interoceptive sensitivity tasks. First, the heartbeat detection task, an IS measure, tested objective accuracy in detecting bodily sensations by asking participants to tap their finger in accordance with their heartbeat. Second, to measure MI, two self-reports measured beliefs about body signals: the Body Sensations Questionnaire (BSQ) and the Physical Concern Index (PCI). Both measures targeted *fear of fear*: the BSQ measured fear of sensations associated with high arousal and panic; the PCI measured reflexive thoughts about physical concerns. The scientists found that PD subjects and controls did not differ in the former (IS), but did differ in the latter (MS), with PD subjects significantly more worried about their somatic sensations than controls. Differences in IS reported between this and other studies likely rely on differences in experimental design and should be further explored. Nevertheless, it is important to note that at least in some PD individuals, MI differences may be more prominent than SI differences.

Alongside these behavioral differences, Cui et al. (2016) explored differences in resting state functional brain connectivity between those with GAD, PD, and controls. They found that both those with GAD and PD had increased positive connectivity between the hippocampus/ parahippocampus and fusiform gyrus compared to controls. Differentiating GAD and PD, those with GAD have decreased connectivity from the post-central gyrus to cuneus and lingual gyrus; middle frontal gyrus to angular gyrus; and the thalamus to posterior cingulate gyrus/ anterior cingulate cortex compared to healthy controls. On the other hand, those with PD exhibit increased connectivity between the post-central gyrus to the thalamus; the right anterior cingulate cortex to the superior temporal gyrus; as well as the right precentral gyrus to the thalamus compared to the healthy controls. Additionally, there is less functional connectivity between the left superior occipital gyrus to the medial temporal lobe in those with PD compared to controls. Cui et al. (2016) suggest the unique increased connectivity from the thalamus to the somatosensory cortex in those with PD relative to controls may explain their heightened interoceptive sensitivity. These findings also show structural differences between those with GAD and those with PD, which may be key biomarkers for distinguishing the two disorders, as well as for potentially distinguishing subtypes of PD.

### Exteroception

In contrast to interoception, which involves internal signals coming from within the body, exteroception refers to how an individual processes information about the physical world. It consists of four categories that go from proximal to distal points: (1) somatic sensation, (2) chemical senses, (3) vision, and (4) audition (Table 1). Somatic sensation, the most proximal sense, has four major modalities, each of which are mediated by distinct systems of receptors and neural pathways, but which all share a common class of sensory neurons: the dorsal root ganglion neurons. However, the neuron terminals are specialized to selectively respond to different stimuli. The first modality reviewed is mechanosensation or discriminative touch, which allows us to sense the size, shape, and texture of objects as well as sense their movement across the skin. Second, proprioception is defined as the sense of the static position and movement of the limbs and body. It should be noted that some researchers separate proprioception from somatosensation, but as it relies on the same dorsal root ganglion pathways as the other somatic senses, we include it here. Conceptually, proprioception can be viewed as an intermediate between internally and externally-referenced sensations: proprioception uses somatic feedback from the joints combined with cues from the vestibular system to represent the position of body in space. This more “proximate sense” uses “the body to describe the external environment and its relation to it” (Quadt et al., 2018). Continuing along the categories of somatic sensation, nociception refers to the signaling of tissue damage or chemical irritation, typically perceived as pain or itch. Lastly thermosensation or temperature sense is the sense of warmth and cold. Chemical senses include olfaction (smell) and gustation (taste). Lastly, the senses of vision and audition are already included in the RDoC as subconstructs of the Perception construct in the Cognitive Systems domain.

The RDoC has already recognized some perceptual categories (primarily vision and audition) and their relevance to mental health. In addition to audition and vision, we have recognized that the RDoC does include a broad Olfaction/Somatosensory/Multimodal Perception subconstruct, however, it does not differentiate between different somatic sensations, nor does it include any units of analysis other than two paradigms — ISI manipulation and smell identification. We propose that olfaction, somatosensory, and multimodal perception be specified separately alongside other perceptual categories. To orient the reader to a more complete taxonomy of perceptual sensations, Table 1 lists several sub-categories of perceptual processing, organized by category and accompanied by a list of associated neural circuits and examples of disorders associated with disruptions in each sub-category of perceptual processing. For brevity, in the next section of this review, we discuss a single example of aberrant perceptual processing for each of these senses in more detail.

### Exteroception and Mental Health

#### Somatic Sensations

##### Mechanosensation in health and ASD

Mechanosensation is the transduction of mechanical forces into neural signals. Several mechanical forces can be sensed in this manner, but typically it refers to touch, which we focus on here in relation to mental health. Mechanosensation interacts with other social, health and cognitive processes: some individuals with ASD are hyper-responsive to sensory stimuli, leading them to avoid touch (Baranek et al., 2013); friendly touch helps typically developing pre-school children delay gratification for eating candy compared to children not receiving friendly touch (Leonard et al., 2014). In fact, the posterior insula seems to be specially tuned to pleasant caressing speeds relayed via tactile C (CT) afferents (Björnsdotter and Olausson, 2011). Interestingly it does not necessarily matter where the source of touch comes from, as demonstrated by Triscoli et al. (2013) who found that those stroked by a person or a robot experienced similar levels of pleasure. Theoretically, it has been proposed that because having one’s skin caressed by a caregiver in early development regularly couples both exteroceptive (i.e., touch) and interoceptive experiences (i.e., reduced physiological arousal), learned predictions regarding these internal and external signals may contribute to the formation of the psychological concept of self (Fotopoulou and Tsakiris, 2017).

Given the role mechanosensation plays in social, health and cognitive processes, it is likely that abnormalities in mechanoreception can lead to problems across the other RDoC domains. For instance, one study by Tavassoli et al. (2016) found a positive correlation between static tactile threshold and ASD symptoms. Furthermore, the researchers found that those with ASD had no difference between static and dynamic thresholds while controls did. Tavassoli et al. (2016) suggested that this difference may be useful as a biomarker for ASD.

##### Nociception and post-traumatic stress disorder (PTSD)

Abnormalities in nociception, or pain processing, may be involved in numerous disorders. Here we focus on PTSD. Individuals may develop PTSD after a traumatic event. Interestingly, there is a high rate of comorbidity between chronic pain and PTSD potentially as a result of excessive peripheral and CNS inflammation present in both conditions (Lerman et al., 2016). In order to distinguish the pathophysiological basis of the comorbidity of chronic pain within PTSD, Lerman and colleagues compared the cerebrospinal fluid concentrations of inflammatory cytokines in combat veterans with and without PTSD following an intramuscular capsaicin injection used to induce pain (2016). They found that both groups experienced an increase in pro-inflammatory cytokines, but only those combat veterans with PTSD experienced an increase in a specific cytokine, IL-1β. On the other hand, an anti-inflammatory cytokine IL-10 increased immediately in the non-PTSD combat veteran group while it was delayed in those with PTSD (Lerman et al., 2016). This altered inflammatory response in the PTSD group corresponded with an altered nociceptive experience: while there was no difference in baseline thermal or mechanical sensitivity between the PTSD and non-PTSD combat veteran groups, following the injection of the capsaicin pain stimulus, the PTSD group showed significantly higher pain intensity and pain unpleasantness ratings than the control group.

##### Thermoception and major depressive disorder (MDD)

Individuals with major depressive disorder (MDD) experience a lower sensitivity to cold and hot stimuli as indicated by their ability to withstand more extreme temperatures (Bär et al., 2005; Terhaar et al., 2010). More research is needed to better understand the relationship between thermoception and MDD. Thus including thermoception in the RDoC may help researchers further involve this dimension in their research.

##### Proprioception and schizophrenia

Proprioception is the perception of the static position of the limbs and body in space. Proprioception plays an important role in motor action, contributing balance and gait. Movement disorders are seen in several clinical disorders, including autism (Donnellan et al., 2013) and schizophrenia (Garvey and Cuthbert, 2017). To understand the etiology of these motor disturbances, it thus may make sense to consider proprioception in the RDoC. In the case of schizophrenia, individuals affected by the disorder also experience reduced proprioception, as evidenced by diminished contralateral gamma and beta activity during a task where the weight of an object abruptly increased (Arnfred et al., 2011). Some researchers argue that in addition to contributing to motor symptoms, abnormalities in proprioception may also lead to difficulty in self-awareness and agency, contributing to the core symptoms associated with schizophrenia (Arnfred et al., 2015). This body of work links sensory sensitivity — in the sense of failures of sensory attenuation — to schizophrenia and misattribution of agency (e.g., Shergill et al., 2005; Joyce et al., 2013). This again emphasizes the importance of sensation in higher cognitive functions and associated psychopathology and the relevance of a sensory processing domain within the RDoC framework.

#### Chemical Senses

##### Olfaction and Alzheimer’s disease

As mentioned previously, olfaction is currently included in the RDoC, but it is currently grouped with somatosensation and multimodal perception and limited detail is included. If a separate sensory domain is added, then olfaction may be moved to this domain instead. Notably, olfactory dysfunction is one of the first observable signs of many disorders, including Alzheimer’s disease (AD) and head trauma (Ansari and Johnson, 1975; Kesslak et al., 1988; Doty et al., 1997). Interestingly, in AD, the severity of olfactory dysfunction was positively correlated with the severity of AD. As we consider the correlation between a growing aging population and the increase in the prevalence of AD, we will need earlier and more precise diagnosis (Dall et al., 2013). Since olfactory testing is relatively inexpensive and identifies symptoms present during the early stages of the disease, it may be one means of early detection and mitigation of further symptoms.

##### Gustation and eating disorders

Gustation is another modality that is currently not covered in the RDoC, yet may be important for understanding some clinical disorders. For example, anorexia nervosa (AN) and bulimia nervosa (BN) are two types of eating disorders which are characterized by extreme eating patterns that may result in medical complications (Westmoreland et al., 2016). Researchers theorize that those with AN and BN have altered taste perception which may contribute to alterations in food consumption (Dazzi et al., 2013). Compared to healthy controls, those with AN and BN had lower perception of bitter taste, but there were no significant differences when sweet or acidic stimuli were tested (Dazzi et al., 2013). Thus, more research on gustation may lead to better understanding of these disorders as well as other disorders.

#### Vision and Audition

Visual and auditory pathways are covered extensively in the RDoC framework in the cognitive domain, and are therefore not a missing sensory feature to be covered within the scope of this paper. We refer individuals to the cognitive systems domain for how these sensory subconstructs are currently reflected in the RDoC.

## Conclusion

### A Sensory Processing and Perception Domain Would Enhance the RDoC

The body and the brain operate as a complex unit, integrating myriad computational processes to coherently guide cognition and behavior. Disruptions in this orchestration are often manifested in mental illness, as demonstrated throughout this paper with regard to disruptions in sensory processing and perception. In certain instances, abnormalities in sensory processing and sensitivity actually lead to impairments in the other domains of functioning and may serve as an early indicator of illness, suggesting that sensory abnormalities may serve as biomarkers for certain mental disorders. Indeed, given the ability to use paradigms from psychophysics to test for sensory disturbances at the individual level, this domain of functioning is an appropriate target for putative biomarkers. Furthermore, the presence (or absence) of sensory abnormalities can differentiate individuals within a specific illness, allowing for the distinction of more homogenous groups, which may aid research. For example, by subgrouping individuals with ASD based on sensory profiles, our understanding of the etiology of various autisms may be less hindered by the inherent heterogeneity within this spectrum disorder. In addition to reducing variance or statistical “noise” in research, such subtyping may lead to more targeted and effective individualized treatment.

Clearly, sensory processing and perception relate to mental health. Given that research can be conducted on constructs outside the RDoC, why do we propose that a sensory domain be added to the RDoC? First, as we have demonstrated throughout this paper, it fulfills the three criteria for a proposed new domain: both sensory processing and the several classes of sensory perception detailed in this review are defined at a (1) psychological as well as (2) biological level, and (3) disturbances thereof relate to clinically-relevant symptoms in several mental health disorders. Aside from fitting these candidate criteria for addition to the RDoC, we argue for the inclusion of sensory processing because it is important that the RDoC be representative of constructs that meaningfully impact mental health. The RDoC framework is a mechanism that informs researchers at all career stages, including early-career researchers, about the current state of the field, including core lines of research at different units of analysis. Additionally, regardless of whether this is actually the case, many believe that the RDoC framework delineates what is considered central to mental health, and therefore aligns with NIMH funding priorities. Because the RDoC can inspire research, it should fully reflect domains that are important to our understanding of mental health, including sensory perception and processing.

We specifically recommend that a new sensory processing domain be added to the RDoC. This domain would include a sensory processing construct, as well as a sensory perception construct. The latter would entail removal of the perception construct from the cognitive systems domain and the addition of interoception, mechanosensation, nociception, thermosensation, proprioception, olfaction, gustation, vision, and audition. While we propose this structure, a group of experts will need to be gathered to develop and finalize the actual framework.

### Principles for Future Development and Use of the RDoC

During the process of weighing the benefits and rationale for proposing the addition of a sensory domain to the RDoC, we have formulated what we see as three important principles for the future development and use of the RDoC.

#### Balance of Inclusivity and Specificity

First, constructs must carefully balance inclusivity and specificity, such that symptoms relevant to several mental disorders are represented within the RDoC (inclusivity) but are able to be described at different levels of neural functioning or “units of analysis” (specificity). This balance enables the functional use of the RDoC by the diverse set of researchers working to understand mental health. For example, when considering disruptions in sensory sensitivity, the symptom literature focuses on a range of behavioral symptoms, including defensiveness, registration, and hyper/hypo-responsivity (Schauder and Bennetto, 2016). While these all have clear behavioral definitions, they currently are not studied across several levels of neural functioning (and indeed, each of these behavioral phenomena may have multiple neurobiological realizations), and therefore are too specific to be included as distinct RDoC constructs.

#### The Need to Study the Intersection of Domains

Second, given the interconnectedness of neural circuits, we see work at the intersection of domains as important for advancing our understanding of everyday functioning; specifying relevant units of analysis for constructs represented in multiple domains is a future challenge for the RDoC framework. Certain behaviors implicate several RDoC domains: for example, phenomenological features of hallucinations relate to several RDoC constructs, with some features relating to multiple constructs — e.g., verbal content relating to both the language and declarative memory constructs in the cognitive systems domain (Ford et al., 2014). Beyond recognizing that several RDoC domains contribute to various symptoms, research paradigms must be designed to investigate the interaction of contributing neural networks. In the case of ASD, a recent special issue of *Developmental Cognitive Neuroscience* recognizes the prevalence of both sensory and social features in ASD, and asks whether they can be integrated (Hamilton and Pelphrey, 2018). Some studies in that issue go on to utilize paradigms that test the interaction of sensory and social processing; for example, Green et al. (2018) use aversive tactile sensory stimuli to modulate social attention in individuals with ASD as a function of the severity of an individual’s sensory over-responsivity. Such integrative approaches are critical to both research and, eventually, treatment. Practically, the RDoC framework must address how paradigms that span multiple domains/constructs and perhaps even multiple units of analysis are to be detailed within the framework.

#### The Promise of Big Data and RDoC 2.0

Finally, the exclusion of sensory and motor processing in the original RDoC matrix may be partially a function of the construction of the RDoC by way of committee. Inclusion of motor and sensory domains would follow a similar process, while other potentially relevant domains might remain missing or underdeveloped. To this end, we would like to briefly highlight big data repositories currently underway as potential empirical avenues for achieving balanced and representative inclusion of psychological constructs related to human mental health and behavior within the RDoC. The NIMH Data Archive (NDA, National Institute of Mental Health, n.d.c) is a set of six data repositories, and includes data and methods from the National Database for Autism Research (NDAR), the RDoC Database (RDoCdb), and Adolescent Brain Cognitive Development (ABCD) study among others. The NDA aims to share human subjects data collected across hundreds of studies in order to accelerate the pace of research. Additionally, the collection of large datasets may one day allow us to take a more data-driven approach in designing a future RDoC. There is considerable interconnectivity between the RDoC domains, especially with respect to certain behaviors and mental health conditions. Creating a taxonomy like that found in the RDoC is difficult. Rather than using our current understanding of the division of mental processes, we would use data to determine the appropriate dimensions to capture the variance in mental functioning in the population across different types of behavior and units of analysis. Given the appropriate large-scale and generalizable datasets, domains and constructs could be identified in an unsupervised (completely data-driven) or partially supervised (perhaps constraining domains but not constructs) manner to determine what actually varies amongst individuals with different mental health conditions. Big data approaches could also incorporate a developmental perspective, indicating what the major domains of importance are at different timepoints (e.g., is motor more important to mental health early in development?).

### The Future of Sensory Processing Research

Moving forward, we are excited about recent and potential changes to the RDoC. We welcome the addition of a motor domain and urge the addition of a sensory domain. Sensory and motor functioning are fundamental to life and we are optimistic that these added domains will improve our understanding of mental health.

## Author Contributions

LH and LA-Z conceived of the topic. LH, AK, and LA-Z developed the framework for sensory processing categories. LH, AK, MW, and LA-Z wrote the review.

## Conflict of Interest Statement

The authors declare that the research was conducted in the absence of any commercial or financial relationships that could be construed as a potential conflict of interest.
